# An anthropometric study of distal tibiofibular syndesmosis (DTS) in a Chinese population

**DOI:** 10.1186/s13018-018-0804-3

**Published:** 2018-04-20

**Authors:** Mingyang Yu, Yao Zhang, Yun Su, Feng Wang, Dewei Zhao

**Affiliations:** 1 Department of Traumatic Orthopedics, Affiliated Zhongshan Hospital of Dalian University, Jiefang Street 6th, Dalian, 116001 China; 2 Department of Orthopedics, Affiliated Zhongshan Hospital of Dalian University, Jiefang Street 6th, Dalian, 116001 China

**Keywords:** Anthropometric, Distal tibiofibular syndesmosis, Chinese

## Abstract

**Background:**

To improve the diagnostic accuracy of distal tibiofibular syndesmoses (DTS), this study quantified the range in variations of the normal DTS in a Chinese population, based on CT scan images.

**Methods:**

The study population comprised 92 patients with unilateral ankle injury. CT scans included the non-injured contralateral DTS. The position of the fibula relative to the fibular notch (incisure) of the tibia was quantified by inclusion or separation indices, based on whether the fibula was within or outside the fibular incisure, respectively. The patients were apportioned accordingly to either a DTS contained- or separate-type group (average ages 45 and 42.1 years, respectively; 19 men/26 women and 24 men/23 women). Further variations in the position of the fibula relative to the tibia were quantified with length, anterior, and posterior indices.

**Results:**

The baseline characteristics of the contained- and separate-type groups were statistically comparable. The length, anterior, extra-anterior, posterior, and extra-posterior indices were successfully calculated. The anterior index of the contained group was significantly greater than that of the separated group, while the posterior index was significantly less.

**Conclusions:**

This study provides measurements of the normal tibiofibular syndesmosis in a Chinese population. In individuals whose fibula lay within the fibular incisure of the tibia, the fibula was likely to be more anterior than that of individuals whose fibula lay outside the incisure. Offered as a reference, these data should improve diagnosis of injury of the DTS.

## Background

Ankle fracture commonly involves injury of the distal tibiofibular syndesmosis (DTS) [1–4], but syndesmosis diastasis is increasingly recognized in sprained ankle as well. Prompt diagnosis and reasonable treatment is important for improving functional outcomes and reducing the risk of early degenerative changes [5].

Injury to the DTS is mainly diagnosed through medical imaging, especially by initial anteroposterior and mortise radiographs. However, some scholars have noted that anatomic variability of the fibula and tibial tubercles can make diagnosis with radiographs difficult [6]. As early as 1985, Sclafani [7] reported that injuries to the ligamentous support of the ankle were subtle and could easily be overlooked on radiograph. Murphy et al. [8] found that medial clear space width on mortise radiographs of the ankle has variability based on the location chosen for measurement and gender. Contralateral radiographic comparison of MCS should be routinely used to identify pathologic widening versus normal anatomic variation.

Harper and Keller [9] later measured the width of the tibiofibular clear space and the tibiofibular overlap, based on carefully positioned anterior-posterior and mortise radiographs of 12 fresh cadavers. They proposed that the width of the tibiofibular clear space, on both anterior-posterior and mortise views, was the most reliable parameter for detecting early syndesmotic widening. Similarly, Pneumaticos et al. [10] determined that the width of the tibiofibular clear space in the anterior-posterior view on plain radiographs was the most reliable parameter for detecting widening of the syndesmosis. However, due to variability among individuals, a view of the contralateral extremity was also warranted in cases of suspected syndesmosis disruption.

Beumer et al. [11] added to the controversy, reporting that radiographic measurements of the DTS have limited application, because there was no standard radiographic parameter to assess syndesmotic integrity. Furthermore, DTS diastasis is often overlooked in cases that do not include an associated fibular fracture.

Computed tomography (CT) is reportedly more sensitive than radiographs for detecting syndesmotic diastasis of ≤ 3 mm [12–16]. However, the accuracy of CT measurements for diagnosis of injuries of the DTS can also be compromised by anatomic variations, and doctors should be aware of the possible differences. For example, Elgafy et al. [17] recently retrospectively reviewed the CT scans of 100 patients with normal DTS and discovered that the fibular incisure was deep or crescent-shaped in 67%, and shallow or rectangular in 33%. Thus, anatomical variations of the fibular borders and the depth of the fibular incisure may interfere in a diagnosis.

To the best of our knowledge, there has been no quantitative study of CT imaging of the normal syndesmosis in the Chinese population. To that end, in the present study, we used CT scans to document the anatomical variety of the normal DTS in a Chinese population. In addition, we argue for the application of inclusion and separation indices to evaluate the anthropometry of the DTS.

## Methods

The Ethics Committee of Dalian University Affiliated Zhongshan Hospital approved this study. The patients provided individual consent.

### Study population

Ninety-two consecutive patients (49 women and 43 men; average age 43.5 years [range, 18 to 82 years]) who attended our hospital between 2014 and 2016 were enrolled in this study. For inclusion, patients had CT scans of both the unilateral ankle injury and the healthy contralateral side. Patients with a history of trauma, inflammatory disease, fracture, or previous surgery on the uninjured side ankle were excluded.

### Materials

A Siemens 64 scanner (Germany) was used to obtain the CT images, with the following parameters: 120 peak kilovoltage, field of view 24 cm, scanning slice thickness 1 mm with reconstruction at 0.625 mm intervals, and rotation time of the tube 0.5 s. An IMPAX/RIS system was used, with vision size 512 × 512 and pixel resolution 0.475 mm. The data of images was exported to a desktop computer in the Dicom format using a secure digital card. The axial CT image was used for index measurements with a RadiAnt Dicom Viewer. The DTS was measured on the CT scans ~ 1 cm proximal to the tibial plafond [18]; this section was 1 mm thick and 9–11 mm proximal to the tibial plafond.

### Measurements

#### Definition and measurement of inclusion index and separation index

The inclusion index is relevant to individuals in whom the fibula is situated within the fibular notch (incisure) of the tibia, while the separation index is a measure that is applicable to individuals in whom the most medial surface of the fibula lies lateral to the most lateral points of the anterior and posterior colliculi of the tibia.

**Fig. 1:**
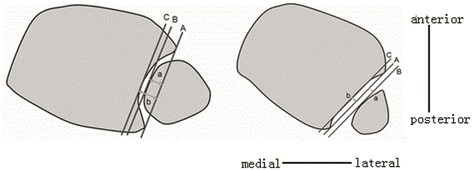
Calculation of the inclusion and separation indices. In each subfigure (**a** and **b**) is shown illustrated cross-sections of the distal tibia (left) and fibula (right). Lines A, B, and C are parallel. Line A represents the tibia incisure plane, drawn tangential to the most lateral aspects of the anterior and posterior colliculi of the tibia. Line B is tangential to the most lateral point of the fibula. Line C is tangential to the most proximal point of the tibia incisure. **a** The inclusion index is the shortest (perpendicular) distance from line A to line B (a), divided by the shortest distance from line A to line C (b), that is, a/b. **b** The separation index is the shortest distance from line A to line B (a), divided by the shortest distance from line A to line C (b), that is, a/b

The measurement process consisted of drawing three parallel lines (A, B, and C) and measuring the distances between those lines (Fig. 1). Line A connects the most lateral aspects of the anterior and posterior colliculi of the tibia and represents the tibia incisure plane. Line B is drawn tangential to the most lateral part of the fibula, and line C is tangential to the bottom of the fibular incisure of the tibia.

The inclusion index is taken to be the shortest (perpendicular) distance from line A to line B (a; Fig. 1a), divided by the shortest distance from line A to line C (b). The separation index is defined as the shortest distance from line A to line B (a; Fig. 1b), divided by the shortest distance from line A to line C (b).

#### Definition and measurement of length index

**Fig. 2:**
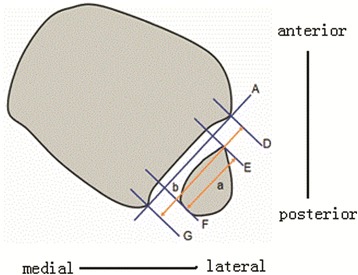
Line A. Lines E and F are tangential to the anterior and posterior borders of the fibula, respectively. The shortest distance between lines E and F (a) is the length of the fibula. Lines D and G are tangential to the anterior and posterior colliculi of the tibia; the shortest distance between lines D and G (b) is the length of the tibia incisure. Finally, the length index is calculated as the length of the fibula (a) divided by the length of the tibia incisure (b), that is, a/b

To determine the length index, again line A is drawn tangential to the two most lateral aspects of the anterior and posterior colliculi of the tibia and represents the tibia incisure plane (Fig. 2). Lines D–G are perpendicular to line A. Lines E and F are tangential to the anterior and posterior borders of the fibula, respectively. The shortest (perpendicular) distance between lines E and F (a) is defined as the length of the fibula. Line D and line G are tangential to the anterior and posterior colliculi of the tibia; the shortest distance between lines D and G (b) represents the length of the tibia incisure. The length index is calculated as the length of the fibula (a) divided by the length of the tibia incisure (b).

#### Definition and measurement of anterior and posterior indices

**Fig. 3:**
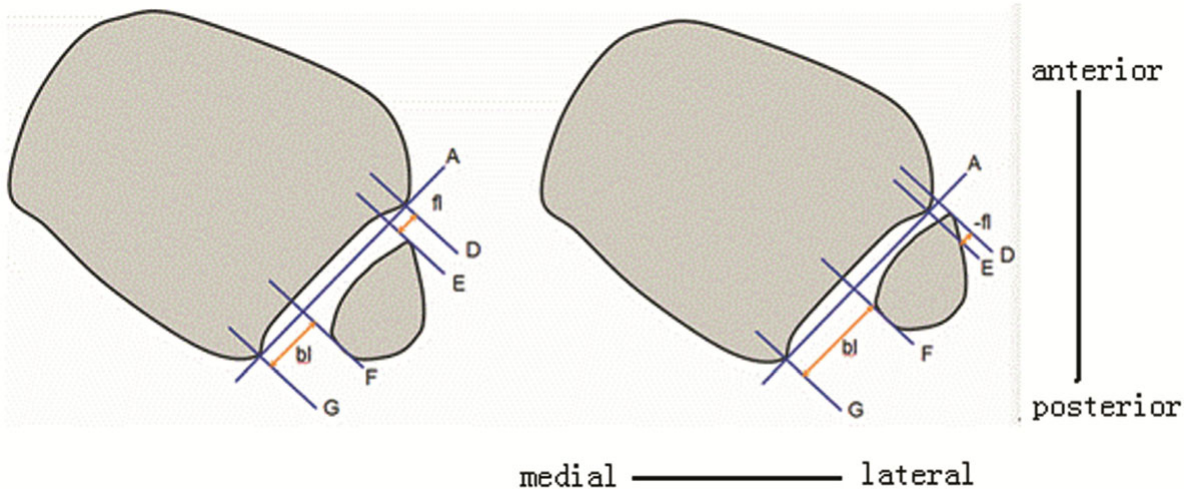
Calculation of the anterior and extra-anterior indices in the centered and relatively anterior fibula. **a** The forward length is defined as the shortest (perpendicular) distance from line D to line E (fl). The backward length is the shortest distance from line F to line G (bl). The anterior index is calculated as the fl divided by the bl (fl/bl). **b** In cases in which the anterior border of the fibula is forward of the anterior colliculi of the tibia, the extra-anterior index is defined as the negative value of fl (−fl), divided by backward length (−fl/bl)

**Fig. 4:**
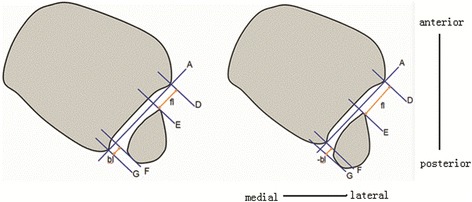
Calculation of the posterior and extra-posterior indices in the relatively posterior fibula. **a** The backward length is the distance from line G to line F (bl) and the forward length is the distance from line E to line D (fl). The posterior index was calculated as backward length divided by forward length (bl/fl). **b** The extra-posterior index was defined as the negative value of backward length (−bl) when the posterior border of the fibula across anterior colliculi of the tibia divided by forward length (−bl/fl)

The anterior, extra-anterior, posterior, and extra-posterior indices were calculated as follows (Figs. 3 and 4). Lines A and D–G are defined as in the preceding sections. Here, the shortest (perpendicular) distance from line D to line E is defined as the forward length (fl), and the shortest distance from line G to line F is the backward length (bl). The anterior index is calculated as the forward length divided by the backward length, or fl/bl. The posterior index is the reciprocal of the anterior index, bl/fl.

The extra-anterior index is defined as the negative forward length value (−fl) when the anterior border of the fibula is anterior to the colliculi of the tibia. The extra-posterior index is defined as the negative backward length (−bl) value when the posterior border of the fibula is posterior to the colliculi of the tibia.

### Statistical analysis

Statistical analyses were performed using an SPSS software package (version 21.0). Analysis of the data was performed by the *t* test. *P* < 0.05 was considered statistically significant.

## Results

The 92 patients were apportioned to two groups, depending on the inclusion and separation indices (Table 1). The demographics of the groups were statistically comparable. Specifically, the patients with a DTS inclusion index were those whose fibula normally resided within the fibular incisure of the tibia. These patients were apportioned to the contained-type group (26 women, 19 men; age range, 18 to 75 years; inclusion index 0.31 ± 0.14). Patients with a DTS separation index were those whose fibula were not within the fibular incisure and were thus considered the separated-type group (23 women, 24 men; ages 19 to 82 years; separation index 0.74 ± 0.71).

**Table 1:** Characteristics of patients with contained type and separate type

	Contained type^a^	Separate type^b^
Gender, M/F, *n*	19/26	24/23
Age, years	45	42.1
Inclusion index	0.31 ± 0.14	–
Separation index	–	0.74 ± 0.71
Length index	0.80 ± 0.08	0.83 ± 0.17
Anterior index	0.49 ± 0.25	0.38 ± 0.20^c^
Extra-anterior index	− 0.52 ± 0.59	− 0.56 ± 0.39
Posterior index	0.52 ± 0.27	0.69 ± 0.37^c^
Extra-posterior index	–	− 0.80 ± 0.34

^a^*n* = 45

^b^*n* = 47

^c^*P* < 0.05, contained-type group compared with the separate-type group

The length index of the contained group (0.80 ± 0.08) was comparable to that of the separated group (0.83 ± 0.17).

Of the 45 patients in the contained-type group, 31 (69%) and 12 (27%) had an anterior index (0.53 ± 0.23) and an extra-anterior index (− 0.52 ± 0.59), respectively. Of the 47 patients in the separated-type group, 23 (49%) had an anterior index (0.38 ± 0.20) and 17 (36%) had an extra-anterior index (− 0.56 ± 0.39). The anterior index of the contained-type group was significantly greater than that of the separated-type group (*P* = 0.012), while the extra-forward indices of the two groups were similar.

Of the 45 patients in the contained-type group, only two (4.4%) had a posterior index (0.52 ± 0.27) and none had an extra-posterior index (Table 1). Of the 47 patients in the separated-type group, 3 (6.4%) and 4 (8.5%) had a posterior index (0.69 ± 0.37) and extra-posterior index (− 0.80 ± 0.34), respectively.

## Discussion

In this study, we successfully characterized contained- and separate-type DTSs by length, anterior, extra-anterior, posterior, and extra-posterior indices in a Chinese population. We found that the anterior index of the contained group was significantly greater than that of the separate group, while the posterior index was significantly less.

**Fig. 5:**
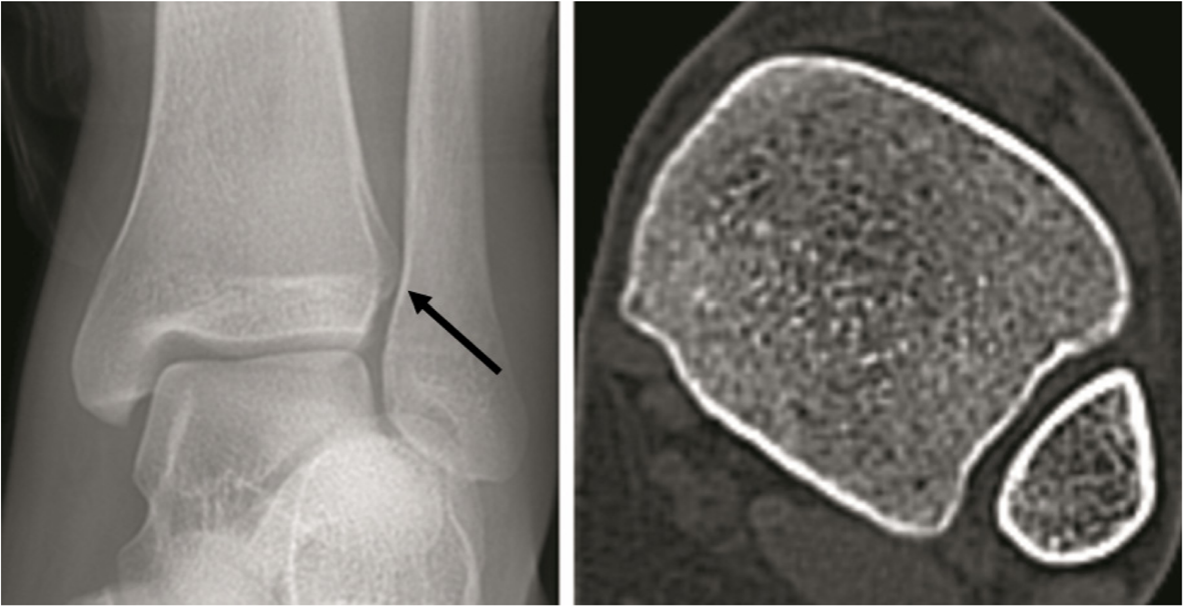
X-ray and CT scans of the DTS with **a** separated left ankle joint and **b** normal left ankle joint. The position indicated by the black tip show the lack of tibiofibular overlap

The tibiofibular overlap is an important measurement for the assessment of the syndesmosis on an AP radiograph. It was difficult to assess on the axial CT section, because the measurement changed with rotation of the foot [17]. In this study, the tibiofibular overlap ≤ 10 mm and talar subluxation have been used to diagnose syndesmosis disruption on radiographs [17]. However, a disadvantage of using X-ray for DTS diagnosis is the possibility of false positive or negative results. Our data suggested that healthy volunteers with rectangular shape of DTS have a relatively long distance of the mid-width of tibia and fibula and wide clear space. It is easy to misdiagnosis a DTS injury on ankle radiographs. These patients are actually normal even if the X-ray seems to indicate a separation (Fig. 5). Though the DTS may be wide in these patients, they do not need to be fixed. In addition, the contained-type DTS could be misdiagnosed when the degree of separation was less significant than the contained index and when tibiofibular was overlapped due to rotation while the DTS was separated. Thus, repeating measurements on multiple radiographs with different views or CT images are recommended.

**Fig. 6:**
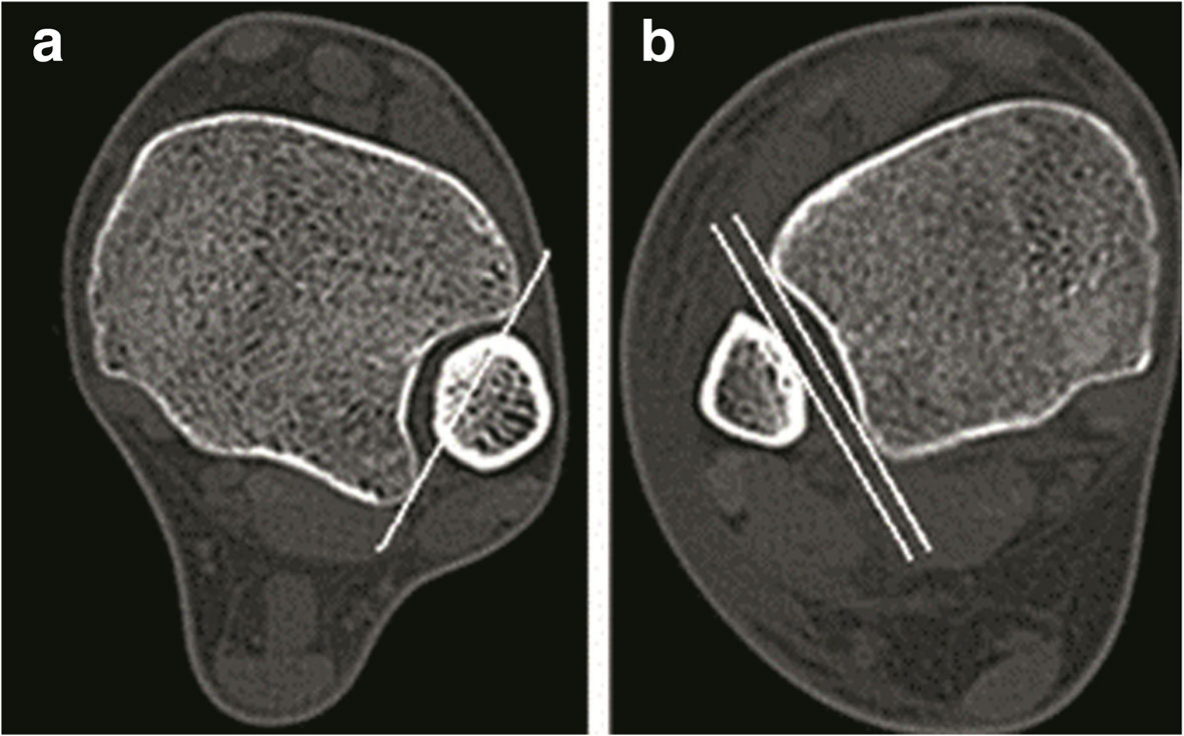
CT scans of representative patients with **a** contained-type and **b** separated-type DTS. **a** The fibula was contained in the fibular incisure. **b** The X-ray can penetrate through the interspace between the fibula and the fibular incisure

Recently, researchers found that CT scans hold the advantage over radiographs for the diagnosis of lesions in the DTS [13, 19] (Fig. 6). Elgafy et al. [17] retrospectively reviewed the shape and measurements of the normal DTS on CT scans of 100 patients and observed that 67% of the fibular incisure was deep, giving the syndesmosis a crescent shape, while 33% of the fibular incisure were shallow, giving the syndesmosis a rectangular shape. Shah et al. [16] found that a lack of overlap combined with a diminutive anterior tibial tubercle on mortise radiograph in some individuals was a normal anatomic variation. On cross-sectional CT imaging, these patients often have a rectangular-shaped syndesmosis as opposed to the more common crescent-shaped syndesmosis. This provides further evidence that individual anatomic variation can affect the diagnosis of DTS, despite the higher sensitivity of CT. Elgafy et al. [17] recommended using these two measurements described in this study as more reliable means to appreciate the degree of the syndesmotic diastasis than using the overlap measurement, which would not be reproducible and accurate if not measured on a precise AP or coronal radiographic view.

Elgafy et al. [17] used CT scans to classify the shapes of the syndesmosis as crescent or rectangular. However, this classification can be subjective and influenced by individual differences, and there has been no standard measurement. In this study, we propose use of inclusion and separation indices, as described herein, for analyzing variation of the syndesmosis. The shape of the syndesmosis can be classified as either contained or separated, based on these indices. We found that 49% of the fibular incisure had an inclusion index of 0.31 ± 0.14, and 51% of the fibular incisure had a separation index of 0.74 ± 0.71. Accordingly, the DTS can be classified as the contained type or the separated type. Since this method is quantitative, it is highly repeatable and not easy to be affected subjectively. It would be helpful to analyze the degree of overlap in clinics.

The diagnosis of a sagittal position shift of the fibula based on lateral and anterior-posterior views is more difficult and easier to be overlooked than that on the horizontal view. This is probably due to the complicated anatomical relationship between the fibular incisure and the fibula, which comprise the main structure of the tibiofibular syndesmosis. Therefore, if we want to use CT to analyze whether the fibula has the displacement of sagittal position, we should determine the change of the relative position between the fibula and the fibular incisure. First, we used the forward and backward indices to determine a sagittal position shift of the fibula. The extra-anterior index is inversed to anterior index, which makes it negative, and likewise for extra-posterior index and posterior index. Second, to analyze the fibula relative to the length of the fibular incisure, we determined the length index. In other words, the clinical doctors should first determine whether the patient is inclusive or dissociative when using the CT images of the horizontal position to analyze whether the fibula has a sagittal position. Additionally, since the location of the fibula is relative to the fibular incisure plane, the fibula may tend to be relatively more anterior of the fibular incisure in individuals with a contained-type DTS and more posterior in those with a separated type. However, when using the length index for diagnosis the sagittal position shift of the fibula, we found that five patients (5.4%) had a fibular length that was longer than the fibular incisure plane. That is to say, these patients used CT for examination, because the margin of the fibula is beyond the anterior and posterior colliculi of the tibia, which increases the difficulty for the diagnosis of sagittal detachment.

One of the limitations of the study is that this study was only conducted in a Chinese population. We are not sure whether there could be a difference between different ethnic groups. Further studies with multi-center collaboration are warranted. Another limitation of CT scans in this clinical setting is an inability to evaluate bone marrow edema and injuries to the ligaments and adjacent soft tissues. Another limitation was there are many scholars who have analyzed the morphology of the syndesmosis; however, these parameters have not been included in our study.

## Conclusions

In conclusion, our study for the first time used CT scans to quantitatively calculate indices that describe normal variations in the anatomical relationship between the fibula and fibular incisure in the Chinese population. This would be helpful for improving the diagnostic accuracy of distal tibiofibular syndesmoses and providing optimal treatment in order to improve functional outcomes and reduce the risk of early degenerative changes for the patients.

## Abbreviations

bl: Backward length
-bl: Negative backward length
CT: Computed tomography
DTS: Distal tibiofibular syndesmoses
fl: Forward length
-fl: Negative forward length
SPSS: Statistic package for social science
